# First-Line Chemotherapy Regimens for Advanced and Metastatic Leiomyosarcoma: Doxorubicin vs. Gemcitabine—A Systematic Review

**DOI:** 10.3390/cancers18020335

**Published:** 2026-01-21

**Authors:** Ilma Khan, Priyal Agarwal, Nassar El Assaad, Ravin Ratan, Elise F. Nassif Haddad

**Affiliations:** 1 Department of Sarcoma Medical Oncology, The University of Texas MD Anderson Cancer Center, Houston, TX 78712, USA; 2 Division of Hematology and Medical Oncology, Medical University of South Carolina, Charleston, SC 29425, USA

**Keywords:** leiomyosarcoma, doxorubicin, gemcitabine, first-line, toxicity

## Abstract

**Background**: Leiomyosarcomas are an aggressive soft-tissue sarcoma that arise from smooth muscle, have a high metastatic potential and account for 10–20% of soft-tissue sarcomas. Despite decades of research, the first-line treatment remains unresolved due to the absence of direct comparative trials, heterogeneous study designs, and trade-offs between efficacy and toxicity. This systematic review evaluates the optimal therapeutic systemic chemotherapy regimens in the first-line setting, specifically gemcitabine- and doxorubicin-based regimens, including associated toxicities. **Methods**: A systematic search in MEDLINE (Ovid), Embase (Ovid), and Cochrane Library (Wiley) identified studies of first-line gemcitabine- or doxorubicin-based chemotherapy for leiomyosarcoma. The review protocol was registered in PROSPERO (CRD420261280028). Of the 3092 articles screened, 11 articles were eligible for inclusion, comprising results from 1225 patients. Eligible studies were in English and included ≥10 patients with advanced/metastatic leiomyosarcoma reporting on LMS-specific outcomes and no prior systemic therapy. This qualitative systematic review synthesizes prospective and retrospective evidence without quantitative meta-analysis. **Results**: The review included two phase 3 trials, six phase 2 trials, one phase 1b trial, and two retrospective studies. While there was no direct comparison in this setting, doxorubicin-based combinations consistently reported higher objective response rates, progression-free survival, and overall survival. The most favorable outcomes were observed in the LMS04 trial with doxorubicin plus trabectedin followed by surgery and trabectedin maintenance, yielding a median progression-free survival of 12 months, overall survival of 33 months, and objective response rate of 36%. This regimen also had the highest grade 3–4 toxicity. **Conclusions**: Doxorubicin-based regimens remain the most active first-line option for leiomyosarcoma, although treatment practices remain heterogeneous.

## Simple Summary

Leiomyosarcoma (LMS) is a rare and aggressive cancer for which the optimal first-line chemotherapy remains uncertain, despite decades of clinical use. This systematic review summarizes the available evidence comparing two commonly used treatment approaches: doxorubicin-based regimens and gemcitabine–docetaxel. Although widely prescribed, these regimens have never been directly compared in first-line clinical trials. Overall, doxorubicin-based combinations—particularly doxorubicin–trabectedin followed by maintenance therapy, and when feasible, surgery—were associated with improved survival outcomes, but at the cost of higher toxicity. In contrast, gemcitabine–docetaxel showed more limited and less durable benefit. This review provides clinicians and researchers with a clear, up-to-date overview of treatment effectiveness, safety considerations, and remaining knowledge gaps, and highlights priorities for future clinical trials in advanced leiomyosarcoma.

## 1. Introduction

Soft-tissue sarcomas (STSs) are a rare, heterogeneous group of mesenchymal tumors comprising over 70 histological types [1]. Leiomyosarcoma (LMS), which originates from smooth muscle, is one of the most common types of STS, accounting for about 10–20% of all STSs [2,3]. Frequent sites include the retroperitoneum, uterus, trunk, and extremities [4]. LMS is broadly classified into uterine (uLMS) and non-uterine soft tissue (STLMS), with distinct molecular and prognostic features [4,5,6]. LMS is an aggressive malignancy with a high metastatic potential and poor long-term outcomes, with a reported 5-year LMS survival rate of ~35% (10–15% for higher grades) [7,8].

Localized LMS is primarily managed with surgical resection, with radiation or chemotherapy added depending on prognostic factors [9]. In contrast, metastatic or unresectable disease requires systemic therapy; most often, cytotoxic agents are aimed at improving survival and symptom burden. Common regimens include doxorubicin-based combinations, gemcitabine (with or without docetaxel), trabectedin, dacarbazine, and ifosfamide [10]. Despite these options, treatment outcomes remain limited, and the optimal first-line regimen has not been clearly established. Due to the absence of head-to-head randomized trials, oncologists must individualize first-line treatment selection in advanced LMS based on indirect evidence, toxicity profiles, and patient-specific considerations rather than clear regimen superiority. Published studies have shown efficacy for both doxorubicin and gemcitabine in the first-line treatment of LMS, making them the central focus of this review [9,11]. Novel targeted therapies and immunotherapies are under investigation, but their role remains uncertain [12,13,14].

Unlike prior reviews that examined LMS across multiple treatment lines or pooled heterogeneous STS populations, this review focuses exclusively on first-line systemic chemotherapy, incorporates newer phase III data such as LMS04, and provides a structured synthesis of efficacy and toxicity within LMS subtypes. Because most studies combine multiple STS subtypes and lack validated LMS-specific analyses, this review focuses exclusively on first-line doxorubicin- and gemcitabine-based regimens, aiming to clarify comparative effectiveness and inform future treatment standards through a focused evaluation of available clinical data.

## 2. Materials and Methods

### 2.1. Research Strategy

This study is a qualitative systematic review conducted in accordance with the Preferred Reporting Items for Systematic Reviews and Meta-Analysis (PRISMA) guidelines; no quantitative meta-analysis was performed due to clinical and methodological heterogeneity [15]. The review protocol was registered in PROSPERO (CRD420261280028). On 19 June 2024, MEDLINE (Ovid), Embase (Ovid), and the Cochrane Library (Wiley) were searched from inception to 18 June 2024. Controlled vocabulary (MeSH and Emtree) and natural language terms for leiomyosarcoma, doxorubicin, and gemcitabine were used, with strategies customized for each database. The search string was “leiomyosarcoma” AND (“gemcitabine” OR “doxorubicin” OR “adriamycin”). Results were limited to English-language publications, with no other restrictions applied.

### 2.2. Inclusion and Exclusion Criteria

The inclusion criteria for each study were as follows: peer-reviewed, clinical trials, prospective and retrospective studies evaluating first-line therapeutic regimens in advanced or metastatic STSs, with at least 10 patients with leiomyosarcoma (LMS) aged ≥ 18 years, for which LMS-specific data was reported, had received no prior systemic therapy, and had been assessed by response evaluation criteria in solid tumors (RECIST) (stable disease, partial response, complete response, and progressive disease) [16,17]. The ≥10 patient cutoff was chosen to avoid anecdotal evidence and allow for the meaningful interpretation of treatment outcomes.

The exclusion criteria for each article were as follows: sarcomas other than LMS; chemotherapy regimens that were not gemcitabine- or doxorubicin-based; articles in languages other than English; duplicate articles; abstracts with no full manuscript; ongoing trials, unless they had been updated with results; and LMS and STS data with unspecified chemotherapy regimens or regimens that were not approved and are not available for prescription. Review articles, editorials, case reports, cases series, and conference abstracts without full data were also excluded.

### 2.3. Data Collection

**Figure 1:**
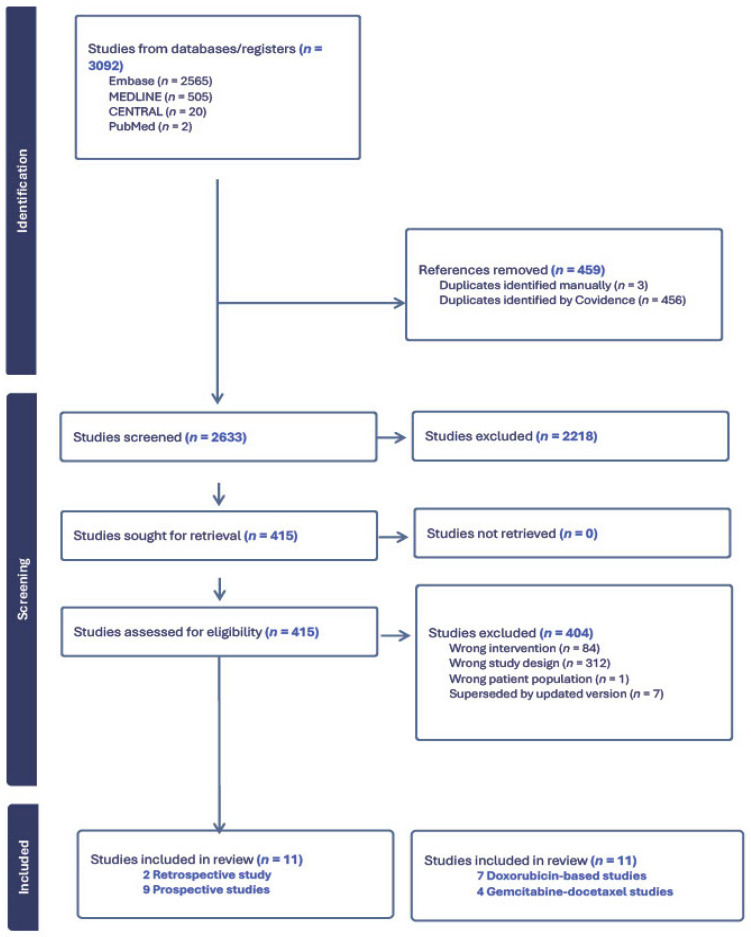
Preferred Reporting Items for Systematic Reviews and Meta-Analyses (PRISMA) flow diagram.

The PRISMA flowchart is shown in Figure 1. A total of 3092 results were retrieved from four databases (MEDLINE = 505; Embase = 2565; Cochrane = 20; PubMed = 2). Two additional studies were manually imported because they were not in the initial search. Of these two studies, SARC002 was not identified in the initial search because it discussed many forms of STSs and did not specifically mention LMS in the title or abstract; however, most patients had LMS, and LMS-specific data were reported [18]. The other study manually imported is the ImmunoSarc II trial, which evaluated doxorubicin, dacarbazine, and nivolumab. This study was published after the researchers had performed their initial screening in Covidence (Veritas Health Innovation, Melbourne, Australia) [19].

These search results were uploaded to Covidence; after deduplication, 2633 unique results were identified for eligibility screening. Once the search results had been retrieved from the databases and uploaded to Covidence, the title and abstracts of all 2633 studies were screened by two independent reviewers (E.N.H. and I.K.). The following parameters were extracted from each article: first author name; publication year; type of study (prospective, retrospective, or review); first-line chemotherapy regimen; study design; trial phase; type of LMS; patient sample size; overall response rate (ORR) according to RECIST; and median progression-free (PFS) and median overall survival (OS) rates as summarized in Table 1. Adverse effects were also compiled for a better comparison of studies and can be seen in Table 2.

### 2.4. PRISMA

The predefined search identified a total of 3092 publications, 2218 of which were excluded after title and abstract screening. A full-text review was conducted using the inclusion and exclusion criteria; 404 articles were excluded because 84 used the wrong intervention, 312 had different study designs; and 1 had the wrong patient population. Seven studies were updates of older trials in this systematic review; thus, the original papers were excluded.

## 3. Results

A total of 11 studies met the inclusion criteria and are summarized in Table 1. These included prospective phase 1b–3 clinical trials and retrospective cohort studies evaluating first-line systemic chemotherapy for patients with advanced or metastatic leiomyosarcoma (STLMS or uLMS). The included studies evaluated two major therapeutic classes:

(1) Doxorubicin-based regimens, including combinations with ifosfamide, dacarbazine, trabectedin, and immunotherapy.
(2) Gemcitabine–docetaxel-based regimens, including combinations with bevacizumab.

Study sample sizes ranged from 26 to 303 patients. Subtype-stratified (uLMS vs. non-uLMS) outcomes were inconsistently reported. Study designs included randomized phase 3 trials (*n* = 2), phase 2 prospective trials (*n* = 6), phase 1b trials (*n* = 1), and retrospective studies (*n* = 2) [18,19,20,21,22,23,24,25,26,27,28].

### 3.1. Doxorubicin-Based Studies

#### 3.1.1. Population and Response Characteristics

Notably, 7 of the 11 studies in this systematic review analyzed the effectiveness of doxorubicin-based chemotherapies as first-line treatment for uLMS and STLMS.

Doxorubicin-based regimens remain the historical foundation of first-line therapy for advanced LMS and represent the earliest prospective evidence, with Sutton et al. (1996) reporting an ORR of 30% and median OS of 9.6 months for doxorubicin–ifosfamide in 35 uLMS patients, providing an early benchmark for anthracycline doublets in this disease [20].

Parallel efforts have explored alternative anthracycline partners. The largest doxorubicin-based study, by D’Ambrosio et al., was a retrospective, multicenter cohort study of three doxorubicin-based regimens in 303 patients with LMS: 117 patients in the doxorubicin and dacarbazine group, 71 in the doxorubicin and ifosfamide group, and 115 in the doxorubicin-alone group. In a propensity-score-matched population (*n* = 205), doxorubicin–dacarbazine achieved the highest efficacy, with ORR 30.9%, median PFS 9.2 months, and median OS 36.8 months. Outcomes were less favorable with doxorubicin–ifosfamide (ORR 19.5%, PFS 8.2 months, OS 21.9 months) and doxorubicin alone (ORR 25.6%, PFS 4.8 months, OS 30.0 months). Six-month PFS rates were 58.2%, 47.1%, and 42.4% for the dacarbazine, ifosfamide, and doxorubicin groups, respectively, confirming superior activity of the dacarbazine combination [24].

Another drug that has shown slight activity in LMS when combined with doxorubicin is cisplatin. A prospective phase 2 trial performed by Edmonson et al. in 2002 focused on doxorubicin, mitomycin, and cisplatin for uLMS. The study had 41 patients, of which 37 were evaluable for toxicity and 35 were evaluable for response. The regimen achieved an ORR of 48% and median OS of 6.3 months, though severe pulmonary toxicity from mitomycin limited its use and warranted caution [21]. Another study that combined doxorubicin with cisplatin was performed in 2015 by Hadoux et al. The researchers retrospectively evaluated doxorubicin–cisplatin–ifosfamide in 38 uLMS patients, of whom 33 were assessable. The regimen achieved an ORR of 48% (12% complete, 36% partial), with median PFS 9.8 months and OS 27 months. At 1, 3, and 5 years, OS rates were 81%, 43%, and 22%, respectively. Twelve patients underwent surgery post-chemotherapy, yielding nine complete and three partial surgical responses [22]. While these regimens demonstrated moderate activity, toxicity concerns and limited uptake place them as exploratory options compared to more established anthracycline doublets.

Subsequent developments in anthracycline combinations incorporated agents with distinct mechanisms of action, notably trabectedin, whose activity in LMS prompted further investigation. Pautier et al. (2015) conducted a prospective, non-randomized, multicenter phase 2 trial targeting uLMS and STLMS with a combination of doxorubicin and trabectedin [23]. The study had 108 evaluable participants and has shown higher activity in uLMS (ORR 59.6%, median PFS 8.2 months, median OS 20.2 months) than STLMS (ORR 39%, median PFS 12.9, and median OS 34.5 months). These observations were later tested in a randomized, multicenter phase 3 trial (*n* = 150, 67 uLMS, 83 STLMS) comparing the combination of doxorubicin and trabectedin, followed by surgical resection if feasible followed by trabectedin maintenance for a year versus doxorubicin alone followed by surgical resection if feasible in the first-line setting for advanced LMS, with randomization stratified by subtype. Patients in the combination arm (*n* = 74) achieved higher efficacy, with ORR 36.5% versus 13.2%, and significantly improved median PFS (12.2 vs. 6.2 months; HR 0.37, *p* ≤ 0.0001) and OS (33.1 vs. 23.8 months; HR 0.65, *p* = 0.0253). In the 2024 update, the doxorubicin–trabectedin arm showed superior outcomes, with a 2-year OS rate of 68% versus 49% for doxorubicin alone, and longer time to second progression (26 vs. 13 months; HR 0.46). Surgery after six cycles was more frequent in the combination group (24% vs. 8%), reflecting greater disease control [25,29].

More recently, immunomodulatory strategies have been introduced into first-line management. The most recent study analyzing first-line doxorubicin-based chemotherapy regimens for uLMS and STLMS was conducted by Martin-Broto et al. in 2024. This prospective phase 1b trial (*n* = 26; 11 uLMS, 15 STLMS) evaluated doxorubicin–dacarbazine–nivolumab as first-line therapy. The regimen achieved an ORR of 56.5%, median PFS of 8.7 months, and a 1-year OS rate of 82%, with median OS not reached, supporting its activity in advanced LMS [19].

#### 3.1.2. Adverse Effects

Across all doxorubicin-based regimens, hematologic toxicities were the most common grade 3–4 adverse events (Table 2). Grade 3–4 hematologic toxicities frequently necessitated dose reductions or treatment discontinuation, limiting real-world feasibility despite observed efficacy. In the Sutton et al. study, the combination of ifosfamide and doxorubicin, which was moderately active but not superior to doxorubicin alone, was associated with grade 3 and 4 neutropenia, with 17 patients affected [20]. When this same combination was compared to the combination of doxorubicin and dacarbazine and with doxorubicin alone in the D’Ambrosio et al. study, 7 out of the 303 patients experienced toxicities that were attributed to ifosfamide, 25 experienced toxicities from their individual treatments, but the most common grade 3 or 4 adverse effects were not mentioned [24]. These studies made it evident that there are relevant toxicities associated with ifosfamide; thus, the drug should be used with caution when treating patients with LMS.

Cisplatin-based regimens are more toxic: Edmonson et al. reported severe leukopenia (89%), thrombocytopenia (81%), and pulmonary toxicity with two treatment-related deaths, while Hadoux et al. observed frequent myelosuppression (neutropenia 74%, thrombocytopenia 60%, anemia 55%), high transfusion rates, hospitalizations, and one toxic death [21,22].

In the phase 2 trial of Pautier et al., doxorubicin–trabectedin was active but associated with high rates of neutropenia (78%), thrombocytopenia (37%), and elevated liver enzymes (39%). Less than 15% of patients stopped treatment due to toxicity, and about 37% had a dose reduction due to thrombocytopenia [23]. The combination regimen was more toxic than doxorubicin alone in the LMS04 trial, with a higher incidence of adverse events and dose reductions. In the doxorubicin-alone group, 56% of patients reported grade 3 or 4 adverse events, compared with 97% in the combination group. The percentages of patients with neutropenia (13% vs. 80%), anemia (5% vs. 31%), thrombocytopenia (0% vs. 47%), and febrile neutropenia (9% vs. 28%) were significantly higher in the combination therapy group. Serious adverse events were more frequent in the combination group (37 vs. 20). No treatment-related deaths occurred with the combination, while one death from cardiac failure was reported in the doxorubicin arm. Although the combination therapy yielded more adverse events, the time to second disease progression was longer (26 months vs. 13 months), making it more active against LMS (HR 0.46) [25,29].

The ImmunoSarc II reported mostly grade 1 or 2 toxicities, including anemia (66.7%), neutropenia (50%), leukopenia (41.7%), and thrombocytopenia (16.7%), with occasional grade 4 neutropenia (17%) and thrombocytopenia (8%) [19].

### 3.2. Gemcitabine-Based Studies

#### 3.2.1. Population and Response Characteristics

Notably, 4 out of the 11 studies evaluated gemcitabine–docetaxel (GD)-based chemotherapy as first-line treatment for patients with uterine and soft-tissue leiomyosarcoma. The earliest trial was not specific for first-line treatment, but half of the patients did not have any prior line of therapy. The earliest was the SARC002-randomized phase 2 trial by Maki et al. (*n* = 122; 119 evaluable), comparing gemcitabine alone (*n* = 49) with GD (*n* = 73) in metastatic STS. LMS patients comprised 9 (18%) in the gemcitabine arm and 29 (40%) in the GD arm. For LMS, partial responses occurred in 1/9 (11%) with gemcitabine and 5/29 (17%) with GD. Disease control at 24 weeks was achieved in 27% vs. 32%, respectively. Median PFS was 6.2 months with GD vs. 3 months with gemcitabine, and median OS was 17.9 vs. 11.5 months (P superiority = 0.97). These findings suggest GD provides clinically relevant improvements in disease control, PFS, and OS compared with gemcitabine alone, supporting its role as a more active regimen in LMS [18].

Additional prospective evidence came from a phase 2 study, performed by Seddon et al., of 44 unresectable LMS patients, in which gemcitabine–docetaxel produced a 25% ORR and 36.4% confirmed stable disease. Median PFS was 7.1 months and OS 17.9 months, with 3- and 6-month PFS rates of 70.5% and 59.1%. The clinical benefit rate was 61.4% [28].

More focused evidence in uterine LMS came from the prospective phase 2 trial by Hensley et al., which evaluated GD as first-line treatment for metastatic uLMS in 42 patients. The regimen achieved an ORR of 35.8% (4.8% complete, 31% partial), with a median response duration of 6 months. Progression-free rates were 59.5% at 12 weeks and 40.5% at 24 weeks. Median PFS was 4.4 months and median OS 16.1 months, supporting GD as an active first-line option in uLMS [26].

The role of GD was further evaluated in the double-blind randomized phase 3 trial by Hensley et al. (2015), comparing GD plus bevacizumab (*n* = 53) versus GD plus placebo (*n* = 54). Median PFS was longer in the placebo group (6.2 vs. 4.2 months; HR 1.12, *p* = 0.58), as was OS (26.9 vs. 23.3 months; HR 1.07, *p* = 0.81). ORR was 31.5% in the placebo arm (median response 8.6 months) and 35.8% in the bevacizumab arm (median response 8.8 months). Stable disease occurred in 31% vs. 32% of patients, respectively. The trial was closed at interim analysis for futility, indicating no benefit from adding bevacizumab [27].

#### 3.2.2. Adverse Effects

Toxicities associated with GD regimens were predominantly hematologic, consistent with their known myelosuppressive profiles (Table 2). In the Maki et al. study, cytopenias were the main grade 3–4 toxicities: neutropenia occurred in 28% with gemcitabine vs. 16% with GD, and thrombocytopenia in 35% vs. 40%. Dose reductions were required in 26% vs. 46%, and treatment discontinuation due to non-hematologic toxicity was higher with the combination (40% vs. fewer in gemcitabine alone). Overall, thrombocytopenia affected 38% of patients, while grade 3 fatigue was more frequent with GD (25% vs. 10%). In Hensley et al. (2008), myelosuppression was prominent: grade 3/4 neutropenia in 17% of patients, grade 3 anemia in 24%, grade 3/4 thrombocytopenia in 14.5%, with 43% requiring red blood cell transfusions and 5% platelet transfusions. Grade 3 fatigue occurred in 17%, and rare severe events included one case of grade 4 hypoxia and one grade 3 allergic reaction [26]. In Seddon et al., anemia (95%), alopecia (88%), and thrombocytopenia (71%) were frequent but mostly grade 1–2. High-grade events included fatigue in 30%, anemia in 24%, and neutropenia in 12%. Eighteen percent discontinued early, and fewer than half completed treatment, confirming the regimen’s substantial toxicity [28].

## 4. Discussion

### 4.1. Cytotoxic Efficacy

When looking at the different chemotherapy regimens summarized in this review, while robust head-to-head comparisons are limited, both doxorubicin- and gemcitabine-based regimens demonstrate clinically meaningful activity, and the available evidence does not support the unequivocal superiority of one strategy across all patients.

First-line treatment of metastatic or unresectable uLMS and STLMS with doxorubicin and trabectedin, followed by trabectedin maintenance, showed significant activity, with the longest prospectively recorded median OS and PFS durations [25,29].

These findings have generated considerable debate in the sarcoma community since patients in the LMS04 trial were allowed to undergo surgery after 6 cycles to resect residual disease, which occurred more frequently in the combination arm. In addition, the maintenance chemotherapy with trabectedin likely contributed to the improved PFS and OS—consistent with findings from the T-DIS-randomized trial, which demonstrated the benefit of continued trabectedin exposure rather than discontinuation. This regimen also comes with increased toxicity, specifically thrombocytopenia and myelosuppression, leading to a high rate of dose reductions, delays, and treatment discontinuation. Notably, LMS04 remains the only prospective study to report an OS benefit of a first-line regimen in a histology-specific trial of STS, whereas all other included trials assessed survival in mixed STS populations without LMS-specific OS estimates [25,29,30]. While LMS04 provides the only LMS-specific prospective OS data, outcomes across studies varied substantially by design, population, and endpoints, underscoring the limitations of cross-trial comparison. 

Other anthracycline-based combinations have also shown activity, though with variable benefits. Therefore, based on the D’Ambrosio et al. retrospective study, doxorubicin and dacarbazine combination remains the most promising and widely prescribed alternative first-line treatment for advanced LMS. Overall, doxorubicin and ifosfamide is one of the most toxic and less effective regimens reported in this setting. While single-agent ifosfamide has limited activity in soft-tissue LMS, likely explaining the disappointing response rates, it does have single-agent activity in uterine sarcomas, and some centers still use doxorubicin and ifosfamide in uLMS [20,24,31,32]. Additionally, there remains a lack of real-world toxicity and efficacy data for the doxorubicin–trabectedin regimen to help position this regimen outside of clinical trials [22,24]. Doxorubicin-based therapy remains the most active and most consistently effective first-line approach.

Gemcitabine–docetaxel gained traction in uterine LMS after earlier phase II trials by Seddon et al. and Dickson et al. demonstrated that GD-based regimens can induce objective responses in LMS; however, both studies reported a limited durability of disease control and high rates of treatment discontinuation, reinforcing that GD is more difficult to manage than doxorubicin in the first-line setting. The GeDDiS phase 3 trial, the only randomized comparison of GD versus doxorubicin, further confirmed that GD does not improve ORR, PFS, or OS compared with doxorubicin. Nearly half of all patients in GeDDiS had LMS, making the findings highly applicable. Toxicity also favored doxorubicin monotherapy, which required fewer dose modifications [11,18,26,27]. These results confirm that GD should not replace doxorubicin as first-line standard therapy but remains a reasonable option for patients unfit for anthracyclines or in subsequent lines of treatment.

Taken together, the available evidence suggests broadly comparable first-line efficacy for doxorubicin- and gemcitabine-based approaches at the population level, with combination anthracycline regimens showing higher activity in select settings but without consistent confirmation across heterogeneous study designs.

### 4.2. Treatment Tolerability

Patient-related factors such as performance status, comorbidities, prior pelvic irradiation, and baseline marrow reserve play a critical role in determining treatment tolerance. In this context, gemcitabine–docetaxel continues to represent a pragmatic alternative for selected patients who are unfit for anthracyclines or in whom cumulative cardiotoxicity is a concern. However, despite its widespread use, particularly in uterine LMS, the available randomized and prospective data consistently demonstrate that gemcitabine-based regimens do not outperform doxorubicin-based therapy in terms of progression-free or overall survival. The relatively modest durability of disease control observed with gemcitabine–docetaxel further underscores its role as a secondary rather than preferred first-line option [25,29,30].

In practice, tolerability considerations often drive regimen selection as strongly as efficacy. Doxorubicin-based combinations, particularly regimens incorporating trabectedin or ifosfamide, are associated with substantial hematologic toxicity and frequent dose modifications, whereas gemcitabine–docetaxel is also myelosuppressive and may be challenging to deliver continuously due to fatigue and cytopenias. Accordingly, the treatment choice frequently reflects a balance between expected benefit and the feasibility of safely delivering therapy with adequate dose intensity.

### 4.3. Patient Selection and Disease Heterogeneity

An additional layer of complexity arises from the biological heterogeneity between uterine and non-uterine leiomyosarcoma. Several studies included in this review suggested differential response patterns between these subtypes, with uterine LMS historically perceived as more chemosensitive, particularly to gemcitabine-based regimens. However, recent data, most notably from LMS04, challenge this paradigm, demonstrating the robust activity of doxorubicin–trabectedin across both uterine and soft-tissue LMS. These findings support the notion that histology alone may be insufficient to guide first-line therapy selection and highlight the need for biologically informed stratification strategies [25,29].

The integration of surgery following systemic therapy, as permitted in the LMS04 trial, further complicates the interpretation of survival outcomes. Surgical resection of residual disease likely contributed to prolonged disease control and overall survival in a subset of patients, particularly in the combination arm. While this multimodal approach reflects real-world sarcoma management, it also introduces a potential confounding effect when attributing benefit solely to systemic therapy. Future trials should prospectively account for the role of surgery and explicitly define its contribution to long-term outcomes [25,29].

Beyond histologic subtype, disease burden and functional status are key determinants of clinical benefit and tolerability. Patients with high tumor burden, rapidly progressive disease, or borderline performance status may require regimens that maximize early disease control while remaining deliverable without prolonged interruptions. These considerations further underscore the individualized nature of first-line selection in LMS.

### 4.4. Emerging Therapies and Future Directions

Importantly, the evidence base includes both randomized and retrospective data sources. Randomized trials offer higher internal validity and reduce confounding, but their strict eligibility criteria may limit generalizability. In contrast, retrospective studies provide real-world insights into regimen utilization and outcomes at the cost of selection bias and heterogeneity. This distinction is particularly relevant when interpreting outcomes across LMS04, GeDDiS, and retrospective cohorts such as D’Ambrosio et al.

Although targeted therapies and immunotherapy are areas of growing interest, cytotoxic chemotherapy remains the standard first-line approach in advanced LMS, with most novel agents evaluated in later-line or investigational settings.

Looking ahead, the emergence of immunotherapy-based combinations represents a promising frontier in the first-line treatment of leiomyosarcoma. The encouraging activity observed with doxorubicin–dacarbazine–nivolumab, including the highest objective response rate reported to date for a doxorubicin-based regimen, suggests that immune modulation may enhance chemotherapy efficacy in biologically selected patients. As compared with other soft-tissue sarcoma subtypes, leiomyosarcoma exhibits increased expression of genes related to antigen presentation and T-cell-mediated immunity, supporting a strong biologic rationale for immunotherapy in this disease [9]. Accordingly, several ongoing trials—including combinations of doxorubicin–pembrolizumab, gemcitabine–pembrolizumab, and retifanlimab with gemcitabine–docetaxel—are actively evaluating the role of immunomodulation in the first-line setting [33,34,35]. Ultimately, advancing the field will require adequately powered, leiomyosarcoma-specific randomized trials that directly compare active regimens, incorporate translational endpoints, and prioritize clinically meaningful outcomes beyond response rates alone.

Several first-line soft-tissue sarcoma trials investigated novel combinations with doxorubicin with olaratumab, palifosfamide, evofosfamide, and conatumumab. Although LMS represented a substantial subset in these studies, none reported LMS-specific efficacy and therefore were not included in quantitative synthesis. Their collective results demonstrate a recurring pattern: investigational combinations produced modest increases in response rates (rising from ~12–20% to ~18–28%) without translating into meaningful or reproducible improvements in PFS or OS, which consistently remained around ~4–6 months and ~15–19 months, respectively [36,37,38,39].

The concurrent development of anthracycline–immunotherapy combinations, as well as newer agents such as lurbinectedin, suggests that the first-line landscape for LMS may evolve significantly in the coming years. Given the strong biologic rationale for immunotherapy in LMS, particularly LMS’s enrichment in antigen presentation pathways and tumor-associated macrophages, trials combining cytotoxic chemotherapy with PD-1 inhibitors or targeting the CSF-1 axis represent promising avenues [11]. Several collaborative efforts are underway to design phase 3 trials to compare either GD with doxorubicin–trabectedin in uLMS, or doxorubicin–dacarbazine with doxorubicin–trabectedin with or without maintenance. These initiatives reflect a growing consensus that a definitive LMS-specific randomized trial is urgently needed to align practice patterns and establish a clear first-line standard. Taken together, these data suggest that the first-line treatment landscape for leiomyosarcoma is likely to evolve substantially in the coming years.

While immunotherapy has shown limited activity in unselected LMS, emerging data suggests that biomarker-enriched strategies may better identify subsets more likely to derive benefit, warranting further investigation in prospective trials.

### 4.5. Limitations

The findings of this systematic review should be interpreted considering several limitations. First, only a few studies have evaluated first-line gemcitabine- or doxorubicin-based chemotherapy regimens specifically in LMS; most of the available evidence comes from broader STS trials in which LMS was analyzed as a subgroup. Even fewer randomized phase III studies have evaluated first-line treatment specifically in LMS, limiting the strength of direct, high-level evidence. Second, no study has specifically compared outcomes of GD and doxorubicin-based combination in the first line setting for LMS. Third, sample sizes across trials, particularly prospective studies, were small, reflecting the rarity of LMS but limiting statistical power and the ability to evaluate important subgroups such as uterine versus non-uterine LMS. Fourth, there was substantial heterogeneity in study design, including differences in prior treatments, eligibility criteria, treatment dosing, response assessment intervals, and definitions of progression, complicating cross-trial comparisons. Fifth, access to trabectedin, supportive care capacity, and local practice patterns differ internationally, which may affect the generalizability of combination regimens in real-world settings. Sixth, the included trials lacked biomarker-driven or molecularly stratified analyses, limiting the ability to identify patient subsets most likely to benefit from specific first-line regimens. Seventh, as with all systematic reviews, the findings may be influenced by publication bias; studies with negative or inconclusive results may be underreported. Finally, although multiple databases were searched, expanding the search to additional engines (such as clinical trial registries, meeting abstracts repositories, and regional databases) may have identified additional unpublished or non-peer-reviewed studies.

## 5. Conclusions

Doxorubicin-based therapy continues to represent the most evidence-supported first-line approach for advanced or metastatic leiomyosarcoma, with the combination of doxorubicin and trabectedin demonstrating the strongest prospective activity reported to date. Gemcitabine–docetaxel remains a reasonable alternative for patients who are unable to receive anthracyclines; however, it has not shown superiority in the limited randomized comparisons available. For clinicians, doxorubicin-based therapy should remain the reference first-line option, with gemcitabine-based regimens reserved for selected patients who are unfit for anthracyclines or in whom cumulative cardiotoxicity is a concern. Advancing treatment for this rare malignancy will require methodologically rigorous studies that directly compare active regimens and better account for biological heterogeneity. Urgent priorities include adequately powered LMS-specific randomized trials, uterine-stratified analyses, and biomarker-driven approaches.

Future research should also explore the potential of immunotherapy combinations, molecularly informed patient selection, and novel biomarker-driven strategies. Together, these efforts are essential to refine first-line standards of care and enable more personalized treatment selection for patients with leiomyosarcoma.

## Figures and Tables

**Table 1:** Study characteristics.

Study	Year	Type of Study	Type of Sarcoma	Regimen	Number of Patients (*n*)	ORR	Median PFS Duration	Median OS Duration
Sutton et al. [20]	1996	Prospective phase 2 trial	Advanced uLMS	Doxorubicin and ifosfamide	35	30.3%; *n* = 10/35 PR—9 CR—1 SD—17	Median duration of response: 4.1 months	9.6 months
Edmonson et al. [21]	2002	Prospective phase 2 trial	uLMS	Doxorubicin, mitomycin and cisplatin	37 (35 evaluable)	23%; *n* = 8/35 PR—5 CR—3 SD—14	Not reported	6.3 months
Hadoux et al. [22]	2015	Retrospective monocentric study	Metastatic and locally advanced uLMS	Doxorubicin, cisplatin and ifosfamide	38 (33 evaluable)	48%; *n* = 16/33 PR—12 CR—4 SD—8	9.8 months	27 months
Pautier et al. [23]	2015	Prospective non-randomized, multicenter phase 2 trial	Advanced uLMS and LMS	Doxorubicin and trabectedin	108 47—uLMS 61—LMS	59.6%; *n* = 28/47 PR—28 SD—13 DC—41 39.3%; *n* = 24/61 CR—2 PR—22 SD—32 DC—56	8.2 months 12.9 months	20.2 months 34.5 months
D’Ambrosio et al. [24]	2020	Retrospective multicenter cohort study	LMS	Doxorubicin and dacarbazine, doxorubicin and ifosfamide, or doxorubicin alone	303 117—doxorubicin and dacarbazine 71—doxorubicin and ifosfamide 115—doxorubicin alone	30.9% 19.5% 25.6%	9.2 months 8.2 months 4.8 months *p* = 0.0023 HR 0.72	36.8 months 21.9 months HR 0.65 30.3 months HR 0.66 *p* = 0.2089
Pautier et al. [25]	2024	Prospective randomized, multicenter, open-label phase 3 trial	Locally advanced or metastatic uLMS and LMS	Doxorubicin and trabectedin, followed by trabectedin maintenance vs. doxorubicin alone	150 67—uLMS 83—LMS 76—doxorubicin alone group 74—doxorubicin and trabectedin group	13.2%; *n* = 10/76 36.5%; *n* = 27/74	6.2 months 12.2 months *p* ≤ 0.0001 HR 0.37	23.8 months 33.1 months *p* = 0.0253 HR 0.65 OS at 2 years doxorubicin alone (49%) doxorubicin/trabectedin (68%)
Martin-Broto et al. [19]	2025	Prospective, single-arm cohort phase 1b trial	Advanced LMS and uLMS	Doxorubicin, dacarbazine, and nivolumab	26 11—uLMS 15—LMS	56.5%; *n* = 13/23 PR—13/23 SD—9/23 PD—1/23	8.7 months	Not reached 1-year OS rate was 82%
Maki et al. [18]	2007	Prospective randomized phase 2 trial	Metastatic STS	Gemcitabine and docetaxel vs. gemcitabine alone	122 49—gemcitabine 9—LMS 73—gemcitabine and docetaxel 29—LMS	27%; *n* = 13/49 PR—4/49 SD—26/49 PD—18/49 LMS-specific PR—1/9 32%; *n* = 23/73 CR—2/73 PR—10/73 SD—39/73 PD—18/73 LMS-specific PR—5/29	6.2 months 3.0 months *P* (superiority) = 0.98	17.9 months 11.5 months *P* (superiority) = 0.97
Hensley et al. [26]	2008	Prospective phase 2 trial	Metastatic uLMS	Gemcitabine and docetaxel	42	35.8%; *n* = 15/42 CR—2/42 PR—13/42 SD—11/42 PD—13/42	4.4 months	16.1 months
Hensley et al. [27]	2015	Prospective randomized phase 3 trial	Metastatic uLMS	Gemcitabine, docetaxel, and bevacizumab vs. gemcitabine, docetaxel, and placebo	107 54—gemcitabine, docetaxel, and placebo 53—gemcitabine, docetaxel, and bevacizumab	31.5%; *n* = 17/54 SD—17/54 35.8%; *n* = 19/53 SD—17/54 *p* = 0.69	6.2 months 4.2 months *p* = 0.58 HR 1.12	26.9 months 23.3 months *p* = 0.81 HR 1.07
Seddon et al. [28]	2015	Prospective phase 2 trial	Unresectable LMS	Gemcitabine and docetaxel	44	25%; *n* = 11/44 PR—11/44 SD—16/44 PD—10/44	7.1 months	17.9 months

**Table 2:** Adverse effects.

Study and Year	Chemotherapy Regimen	Grade 3 or 4 Events	Grade 5 Events	Grade 3 or 4 Neutropenia	Grade 3 or 4 Febrile Neutropenia	Grade 3 or 4 Liver Toxicity
Sutton et al., 1996 [20]	Doxorubicin and ifosfamide	Granulocytopenia—48.6% (17/34)	5.9% (2/34)	None reported	5.7% (2/34)	None reported
Edmonson et al., 2002 [21]	Doxorubicin, mitomycin, and cisplatin	Leukopenia—89% (33/37) Thrombocytopenia—81% (30/37)	5.4% (2/37)	None reported	None reported	2.7% (1/37)
Hadoux et al., 2015 [22]	Doxorubicin, cisplatin, and ifosfamide	Nausea—11% (4/38) Vomiting—13% (5/38) Asthenia—18% (7/38) Anemia—55% (20/38) Thrombocytopenia—60% (23/38)	2.6% (1/38)	74% (28/38)	37% (14/38)	None reported
Pautier et al., 2015 [23]	Doxorubicin and trabectedin	Thrombocytopenia—37% (40/108) Anemia—27% (29/108) Fatigue—19% (21/180) Leucocytes—76% (82/108)	2.8% (3/108)	78% (84/108)	24% (26/108)	Increased ALT—39% (42/108)
D’Ambrosio et al., 2020 [24]	Doxorubicin and dacarbazine, doxorubicin and ifosfamide, or doxorubicin alone	None reported	None reported	None reported	None reported	None reported
Pautier at al., 2024 [25]	Doxorubicin alone	Anemia—12% (9/75) Leukopenia—5.33% (4/75)	Cardiac failure −1.33% (1/75)	16% (12/75)	9.33% (7/75)	Increased ALT—1% (1/75) Increased AST—1% (1/75)
	Doxorubicin and trabectedin	Anemia—51.35% (38/74) Leukopenia—98.6% (73/74) Thrombocytopenia—60.81% (45/74)	0	Cycle 1 to 6—77.02% (57/74) Cycle 6 to progression—41.9% (31/74)	21.62% (16/74)	Increased ALT—53% (39/74) Increased AST—16% (12/74) Increased ALP—4% (3/74) Increased bilirubin—11% (8/74)
Martin-Broto et al., 2025 [19]	Doxorubicin, dacarbazine, and nivolumab	Anemia—20.8% (5/23) Leukopenia—25% (6/23)	0	33.4% (8/23)	4.2% (1/23)	None reported
Maki et al., 2007 [18]	Gemcitabine alone	Hemoglobin—13% (6/49) Thrombocytopenia—35% (17/49)	0	28% (14/49)	7% (3/49)	None reported
	Gemcitabine and docetaxel	Hemoglobin—7% (5/73) Thrombocytopenia—40% (29/73)	0	16% (12/73)	5% (4/73)	None reported
Hensley et al., 2008 [26]	Gemcitabine and docetaxel	Leukopenia—14% (6/42) Thrombocytopenia—14% (6/42) Anemia—24% (10/42)	0	16.7% (7/42)	None reported	Elevated AST or ALT—2% (7/42)
Hensley et al., 2015 [27]	Gemcitabine, docetaxel, and placebo	Anemia—33% (17/51) Thrombocytopenia—28% (15/51)	0	23% (12/51)	None reported	None reported
	Gemcitabine, docetaxel, and bevacizumab	Anemia—13% (7/52) Thrombocytopenia—36% (19/52).	0	22% (12/52)	None reported	None reported
Seddon et al., 2015 [28]	Gemcitabine and docetaxel	Anemia—24% (10/42) Thrombocytopenia—7% (3/42) Fatigue—31% (13/42) Dyspnea—17% (7/42) Infection—12% (5/42)	0	12% (5/42)	None reported	None reported

## Abbreviations

The following abbreviations are used in this manuscript:

LMS	Leiomyosarcoma
STS	Soft-tissue sarcoma
GD	Gemcitabine–docetaxel
STLMS	Non-uterine soft tissue
uLMS	Uterine soft tissue
